# A randomized controlled trial of qigong for fibromyalgia

**DOI:** 10.1186/ar3931

**Published:** 2012-08-03

**Authors:** Mary Lynch, Jana Sawynok, Chok Hiew, Dana Marcon

**Affiliations:** 1 Departments of Anesthesiology, Psychiatry and Pharmacology, Dalhousie University, QEII Health Sciences Centre, 4th floor, Dickson Centre, 5820 University Avenue, Halifax, NS B3H 1V7, Canada; 2 Department of Pharmacology, Dalhousie University, 5850 College Street, Halifax, NS B3H 4R2, Canada; 3 330 Woodbridge Street, Fredericton, NB, Canada; 4 6178 Quinpool Road, Halifax, NS, Canada

## Abstract

**Introduction:**

Fibromyalgia is difficult to treat and requires the use of multiple approaches. This study is a randomized controlled trial of qigong compared with a wait-list control group in fibromyalgia.

**Methods:**

One hundred participants were randomly assigned to immediate or delayed practice groups, with the delayed group receiving training at the end of the control period. Qigong training (level 1 Chaoyi Fanhuan Qigong, CFQ), given over three half-days, was followed by weekly review/practice sessions for eight weeks; participants were also asked to practice at home for 45 to 60 minutes per day for this interval. Outcomes were pain, impact, sleep, physical function and mental function, and these were recorded at baseline, eight weeks, four months and six months. Immediate and delayed practice groups were analyzed individually compared to the control group, and as a combination group.

**Results:**

In both the immediate and delayed treatment groups, CFQ demonstrated significant improvements in pain, impact, sleep, physical function and mental function when compared to the wait-list/usual care control group at eight weeks, with benefits extending beyond this time. Analysis of combined data indicated significant changes for all measures at all times for six months, with only one exception. *Post-hoc *analysis based on self-reported practice times indicated greater benefit with the per protocol group compared to minimal practice.

**Conclusions:**

This study demonstrates that CFQ, a particular form of qigong, provides long-term benefits in several core domains in fibromyalgia. CFQ may be a useful adjuvant self-care treatment for fibromyalgia.

**Trial registration:**

clinicaltrials.gov NCT00938834.

## Introduction

Fibromyalgia is a chronic pain condition characterized by widespread musculoskeletal pain of greater than three months duration, and it is associated with fatigue and sleep disturbance as well as other symptoms [1,2]. It affects approximately two to three percent of the population, and leads to disability, impaired quality of life and increased health care utilization [3,4]. Treatments for fibromyalgia are focused on symptom relief along with teaching patients self-management strategies for living with chronic pain. Pregabalin, duloxetine and milnacipran have been approved by the US Food and Drug Administration for treatment of fibromyalgia [1], but these and other pharmacological agents provide only partial improvement. Psychological approaches [5], exercise [6], and complementary and alternative techniques [7,8] have been evaluated in fibromyalgia, and these also provide only partial effects. The limited effectiveness of single treatments has led to suggestions that multimodal approaches are required [9,10]. Meta-analysis indicates post-treatment benefits of multicomponent treatment, but there is little evidence of efficacy in core domains in the long-term [9]. Many individuals with fibromyalgia continue to experience disabling pain and fatigue over the long-term, and additional approaches are needed.

Recent controlled trials have demonstrated that qigong and tai chi offer benefits in fibromyalgia that persist to four to six months [11,12]. These practices have recently been characterized as "meditative movement", and constitute a distinct category of exercise [13]. Earlier studies evaluating qigong for fibromyalgia involved limited amounts of practice (one hour qigong/week) as part of an 8-week mind-body intervention [14], an 8-week combined education/relaxation/qigong intervention [15] or a 12-week body awareness therapy [16] and reported only modest benefit. In a more recent controlled study, daily qigong self-practice (40 minutes) was encouraged for the duration of the seven-week program, and benefits reported in several domains were maintained at four months [11]. A further pilot trial of qigong self-practice for nine weeks (45 minutes daily) indicated reductions in pain and impact, and improvement in physical function for six months [17].

The current study is a randomized controlled trial designed to compare the effects of self-practice of qigong (45 minutes daily, eight weeks) with a control group over a six-month interval. One hundred participants were assigned to either an immediate training group or a wait-list control group who continued with their usual care. At the end of six months, the wait-list group also received training and constituted a delayed practice group. This study design allows for two separate treatment cohorts to be compared, as well as for consideration of outcomes in a larger combined treatment group.

## Materials and methods

### Participants

This trial was conducted from September 2009 to July 2011 at the Pain Management Unit, Queen Elizabeth II Health Sciences Center, Halifax, Nova Scotia. The Ethics Review Committee of the hospital approved the study protocol. Eligible participants met the 1990 American College of Rheumatology criteria for fibromyalgia [18]; all reported widespread pain bilaterally with pain above and below the waist and axial skeletal pain for three months or longer, as well as at least 11 of 18 tender points. Medications must have been stable for at least 14 days prior to participating, and the average 7-day pain score had to be ≥ 4.0 on an 11-point numerical rating scale. Patients were asked to practice qigong for 45 to 60 minutes per day for eight weeks once training was complete. Participants were excluded if they were already practitioners of qigong or if there was a significant medical disorder that the study physician thought would compromise participant safety.

### Study design

Recruitment occurred primarily in response to advertisements in the local newspaper. A total of 268 subjects responded, 148 were screened by telephone, and 120 underwent clinic screening until 100 participants were available for the study. Participants were assigned, using computer generated numbers, to an immediate qigong training group or to a control group (wait-list/usual care), which subsequently underwent delayed training. Randomized group assignments were sealed in opaque white envelopes. Once participants consented to enter the trial, envelopes were opened and assignments made to an immediate training date or to a delayed training time six months later. Using this design, all participants received qigong training. There were three main training cohorts of 20 to 30 per group, and one additional smaller training group at the end for the third wait-list group.

### Qigong intervention and control group

There are many forms of qigong. The present study examined Chaoyi Fanhuan Qigong or CFQ [19] because it is available locally with community-based trained practitioners, and because CFQ had shown benefits in fibromyalgia in a pilot study [17]. Qigong training consisted of an initial workshop conducted over three consecutive half-days by a qualified CFQ instructor. Participants received training in level 1 CFQ; this consisted of instruction in seven key movements known as "the hexagram" and ancillary exercises. Hexagram movements consist of choreographed movements that emphasize softness, relaxation, downward releases and full body distribution of "qi". Once initial training was complete, participants were asked to practice CFQ at home for 45 to 60 minutes per day for eight weeks; time could be broken up into shorter sessions during the day. Participants returned for a 60 minute weekly review/group practice sessions for these eight weeks. Participants were then encouraged to continue self-practice for six months. Outcomes were assessed via questionnaires filled out by each patient on the day of training (baseline) and at the eight-week review session; four- and six-month outcomes were returned by mail. The control group consisted of a wait-list group that continued on with their usual care. This group completed baseline measures on the day of screening, and returned subsequent outcomes by mail at eight weeks, four and six months. When they returned for CFQ training six months later, they followed the procedure described above.

### Adherence to program

Adherence to the protocol was determined by self-reports on a qualitative questionnaire. This questionnaire was completed at eight weeks, and also at four and six months. The mean (SD) self-reported practice time per week for all participants who completed the trial was 4.9 (1.7) h at eight weeks (*N *= 73), 2.9 (2.1) h at four months, and 2.7 (2.1) h at six months. Approximately 52% of participants (*N *= 38/73) self-reported practice of ≥ 5 h per week at eight weeks, which corresponds to 45 minutes per day; practice times for this group were 6.2 (1.1) h, 3.8 (2.0) h, and 3.3 (2.1) h at respective time intervals. Further information in the qualitative reports will be analyzed and published separately.

### Outcome measures and follow-up

Assessments consisted of outcomes in several core domains in accordance with recommendations of the Initiative on Methods, Measurement, and Pain Assessment in Clinical Trials (IMMPACT) [20]. Pain was the primary outcome, and was measured using an 11-point numerical rating scale for pain intensity (NRS-PI) with anchors "no pain" and "pain as bad as you can imagine". Numerical rating scales have been widely used in pain research, and have been demonstrated to identify clinically meaningful changes [21].

Secondary outcome measures included the Fibromyalgia Impact Questionnaire (FIQ), Pittsburg Sleep Quality Index (PSQI), and SF-36 Health Survey. The FIQ is a 10-item validated scale that assesses physical functioning, pain, depression, anxiety, fatigue, morning tiredness, stiffness, job difficulty and overall well-being; it has demonstrated reliability and validity [22]. The PSQI is a well-validated and reliable assessment tool for measurement of sleep quality in clinical populations [23]. The SF-36 Health Survey [24] is the most commonly used generic measure of health related quality of life and is recommended in IMMPACT guidelines [20].

### Statistical analysis

A previous trial of qigong for fibromyalgia (seven weeks, follow-up at four months) involved 58 participants (28 to 29 per group) [11], and a more recent trial of tai chi for fibromyalgia over six months involved 66 participants (33 per group) [12]. Both studies showed statistically significant effects on pain, FIQ scores, and physical and psychological health using these sample sizes. Because of the commitment to practice involved in the present trial (self-practice of 45 to 60 minutes daily for eight weeks) and the duration of the trial (six months), whereby there was the potential for a significant attrition, we targeted a trial recruitment of 50 per group for 100 in total.

Statistical analysis focused on change from baseline scores for the primary (NRS-PI), and secondary (FIQ, PSQI, SF-36 Physical and Mental) measures. Comparisons of mean change scores were made between the immediate treatment and wait-list control groups at eight weeks, four months and six months. In order to limit the risk of Type I error, multivariate analysis of variance (MANOVA) was used. Mean change scores for all subjects (combination group) who underwent training and practice were also compared to change scores during the wait-list phase.

To evaluate the extent of clinically meaningful outcomes, the proportion of subjects in each study group who achieved changes from baseline that reached previously established clinical criteria were compared using the Chi square test.

Qigong practice times per week self-reported at Week 8 were categorized into three groups: 1) "per protocol" where practice times were ≥ 5 hours per week, in keeping with the recommended 45 to 60 minutes per day; 2) > 3 but < 5 hours per week; and 3) ≤ 3 hours or less per week. This last group was considered "minimal practice" and served as a basis of comparison with the per protocol group to examine the relationship between practice time and magnitude of change from baseline on each outcome measure; this comparison was made using MANOVA.

## Results

### Baseline characteristics of participants

Table 1 shows baseline characteristics of participants assigned to the immediate practice and wait-list/delayed practice groups. Most participants (94 to 98%) were female. The mean age at enrollment was 52 years, and the mean duration of fibromyalgia was 9.6 years. Several additional pain diagnoses were involved (Table 1); the incidence of these did not differ between groups, except for orofacial pain which was higher in the wait-list/delayed practice group (*P *< 0.007). Participants had previously been treated with several drugs from major analgesic and adjuvant drug groups, and had also tried a variety of complementary and alternative therapies. There was no difference in the analgesic pharmacological profile between groups.

**Table 1:** Study participants and baseline characteristics

Variable	Immediate practice group	Wait-list, then delayed practice group
**Randomized**	*N *= 53	*N *= 47
**Completed study (%)**	*N *= 43 (81.1%)	*N *= 30 (63.8%)
**Gender M:F (% ratio)**	3:50 (6:94)	1:46 (2:98)
**Age at enrollment, years (SD)**	52.81 (8.91)	52.13 (8.56)
**Duration of FM, years (SD)**	9.67 (6.87)	9.62 (7.56)
**Total tender points (IQR)**	15 (14, 16)	15 (13, 16)
**BMI at enrollment (IQR)**	30 (25.4, 34.7)	29.4 (24.8, 34.8)
**Additional pain diagnoses**		
**Back**	23 (43.3%)	21 (44.7%)
**Cervical sprain**	16 (30.2%)	18 (38.3%)
**Diabetic neuropathy**	2 (3.8%)	3 (6.4%)
**Post-surgical/post-traumatic**	4 (7.5%)	8 (17.0%)
**Headache**	31 (58.5%)	27 (57.4%)
**Orofacial pain**	23 (43.4%)	33 (70.2%) *P *< 0.007
**Osteoarthritis**	22 (41.5%)	17 (36.2%)
**Rheumatoid arthritis**	5 (9.4%)	4 (8.5%)
**Other**	16 (30.2%)	13 (27.7%)
**Previous treatments**		
**Anticonvulsants**	14 (26.4%)	25 (53.2%)
**Antidepressants**	45 (84.9%)	34 (72.3%)
**Nerve blocks/injections**	7 (13.2%)	4 (8.5%)
**NSAIDs**	37 (69.8%)	36 (76.6%)
**Opioids**	22 (41.5%)	14 (29.8%)
**Acupuncture**	24 (45.3%)	24 (51.1%)
**Chiropractic**	11 (20.8%)	17 (36.2%)
**Naturopath/Homeopath/Osteopath**	17 (32.1%)	22 (46.8%)
**Massage therapy**	36 (67.9%)	32 (68.1%)
**Physiological therapies**	26 (49.1%)	23 (48.9%)
**Psychological therapies**	19 (35.8%)	20 (42.6%)
**Current pain medications**		
**Anticonvulsants**	13 (24.5%)	14 (29.8%)
**Antidepressants**	20 (37.7%)	15 (31.9%)
**NSAIDs**	26 (49.1%)	27 (57.4%)
**Opioids**	19 (35.8%)	11 (23.4%)
**Other**	29 (54.7%)	28 (59.6%)
**Total number (SD)**	2.43 (1.41)	2.49 (1.59)

BMI, body mass index; IQR, interquartile range; SD, standard deviation

**Figure 1:**
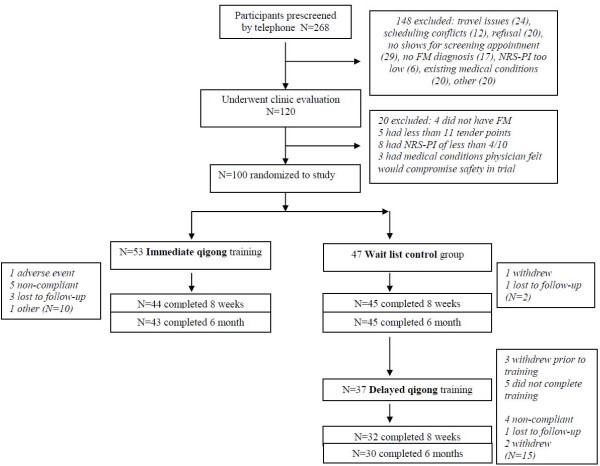
**Screening, randomization and disposition of participants**.

Figure 1 shows the flowchart for disposition of subjects throughout the study. Randomization resulted in assignment of *N *= 53 to the immediate, and *N *= 47 to the delayed group. *N *= 43/53 (81.1%) completed the immediate qigong training protocol for six months, and *N *= 30/37 (81.1%) completed the delayed qigong protocol for six months. *N *= 30/47 (63.8%) completed the wait-list protocol for six months and then the training protocol for six months.

### Pain and impact of fibromyalgia

**Figure 2:**
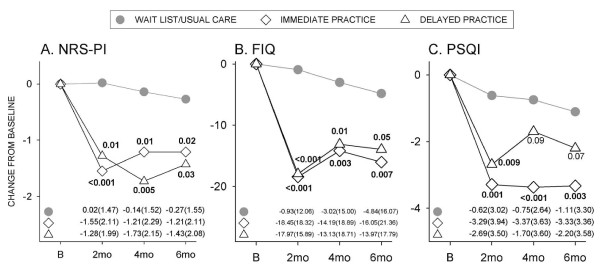
**Effects of CFQ practice on pain, function and sleep**. Change from baseline in (**A**) numerical rating scale-pain intensity (NRS-PI) score, (**B**) fibromyalgia impact questionnaire (FIQ) score, and (**C**) Pittsburgh Sleep Quality Index (PSQI) scores in immediate and delayed qigong practice groups compared to the wait-list/usual care control. Values are means for the (•) usual care, (◇) immediate practice, and (Δ) delayed practice groups. Numbers at bottom of panels indicate mean (SD) values for respective groups. Between-group *P*-values indicated within figure. B, baseline; mo, month.

Table 2 shows baseline values for all outcomes in the immediate practice group, the wait-list group and then the delayed practice group. There were no differences in baseline values in any measure between groups. NRS-PI scores remained relatively stable during the six-month interval in the wait-list group (Figure 2A). There were statistically significant reductions in pain at all times compared to the wait-list/usual care group in both immediate and delayed CFQ practice groups (Figure 2A). Both groups showed similar results despite the six-month delay in training for one group, and this indicates the reproducibility of outcomes in two cohorts. Figure 2B indicates change in FIQ scores from baseline. FIQ scores were reduced by 17 to 18 units from baseline at eight weeks in both the immediate and delayed CFQ practice groups; in both instances this was significantly reduced compared to the control group (*P *< 0.001). While there was a slight rebound in FIQ scores at four and six months, significant differences were maintained in both individual CFQ groups compared to the control group (Figure 2B).

**Table 2:** Baseline values for outcome measures

Baselines Mean (SD)	Immediate practice group	Wait-list group	Delayed practice group
**NRS-PI**	6.45 (1.45)	6.55 (1.06)	6.65 (1.38)
**FIQ**	65.53 (14.44)	61.83 (13.36)	59.73 (15.09)
**PSQI**	13.79 (2.95)	13.09 (3.79)	12.35 (3.76)
**SF-physical**	29.94 (8.30)	32.55 (8.82)	33.22 (8.36)
**SF-mental**	38.13 (9.62)	40.38 (10.08)	39.03 (11.03)

FIQ, fibromyalgia impact questionnaire; NRS-PI, numerical rating scale pain intensity; PSQI, Pittsburgh Sleep Quality Index

### Sleep and physical and mental function

**Figure 3:**
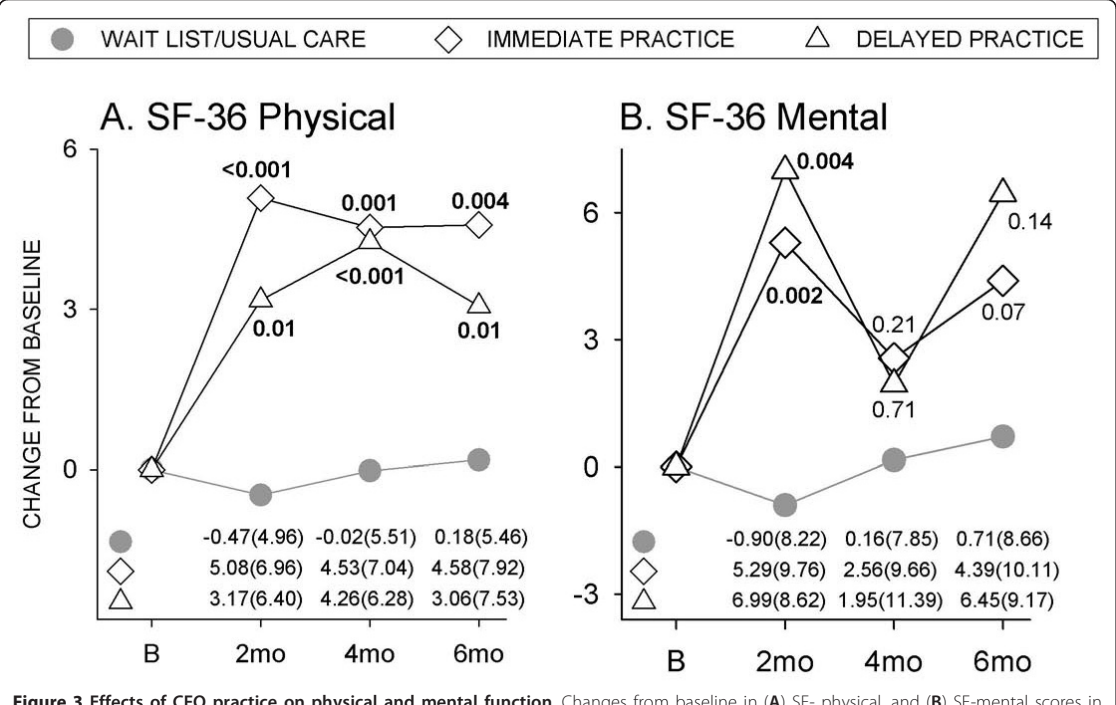
**Effects of CFQ practice on physical and mental function**. Changes from baseline in (**A**) SF- physical, and (**B**) SF-mental scores in immediate and delayed qigong practice groups compared to the wait-list/usual care control. Values are means for the (•) usual care, (◇) immediate practice and (Δ) delayed practice groups. Numbers at bottom of panels indicate mean (SD) values for respective groups. Between-group *P*-values indicated within the figure. B, baseline; mo, month.

Sleep scores showed downward drift over six months in the wait-list group (Figure 2C). PSQI scores were significantly improved in both immediate and delayed CFQ groups at eight weeks; improvements were maintained in the immediate training group, but exhibited rebound in the delayed group. Physical and mental function scores remained relatively stable over six months in the wait-list group (Figure 3A, B). Following CFQ practice, SF-36 physical scores were significantly improved at eight weeks and this was maintained at four and six months in both individual groups (Figure 3A). SF-36 mental scores were also significantly improved in individual CFQ groups at eight weeks, but did not differ from controls at later times (Figure 3B).

### Combined outcomes in two CFQ groups

**Figure 4:**
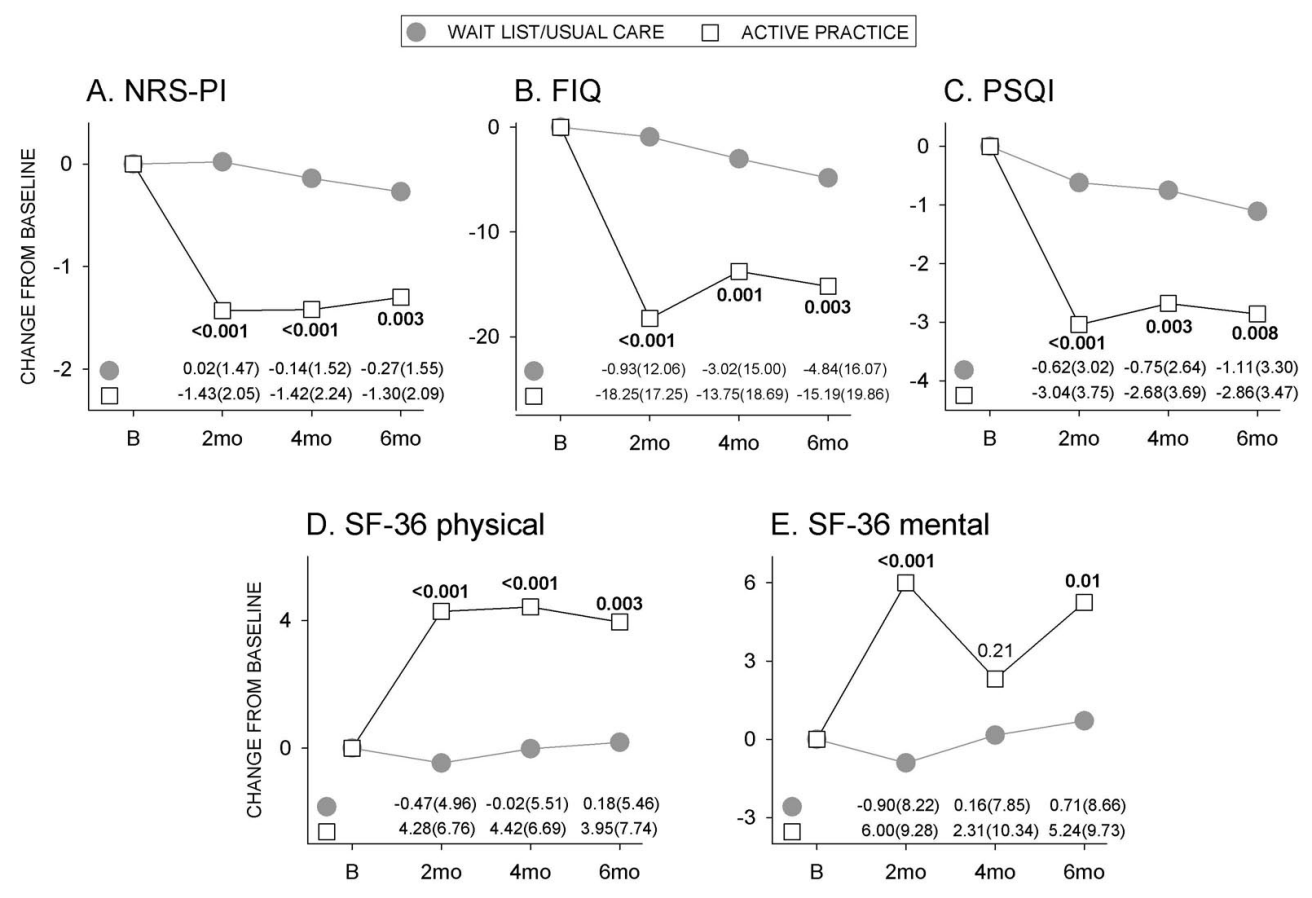
**Mean differences from baseline for all participants who completed CFQ training and practice**. (**A**) NRS-PI, (**B**) FIQ, (**C**) PSQI, (**D**) SF- physical, (**E**) SF-mental scores. Numbers at bottom of panels indicate mean(SD) values for respective groups. Between-group *P-*values indicated within figure. B, baseline; mo, month.

When data for the immediate and delayed practice groups were combined, the significant reductions in pain reported at the end of the eight-week training/practice interval (*P *< 0.001) were sustained in the longer term to four months (*P *< 0.001) and six months (*P *= 0.003) (Figure 4A). With respect to FIQ scores, the combination group exhibited significant improvements compared to the control group at all times (two, four and six months) (Figure 4B). There was also a significant improvement in sleep (Figure 4C) and in physical function (Figure 4D) at all times over the six-month interval in the combination group. Mental function was significantly improved at two and six months, but not at four months (Figure 4E).

The median duration of fibromyalgia in this study was nine years. A *post-hoc *analysis of those above and below the median in the combination CFQ group indicated similar changes in pain in both cohorts (mean NRS-PI reductions of 1.38, 1.52 and 1.35 for < 9 years and 1.49, 1.35 and 1.26 for ≥ 9 years at eight weeks and four and six months, *P *= 0.82, 0.77 and 0.85, respectively). *Post-hoc *analysis of those with fibromyalgia for < 9 or ≥ 9 years indicated no difference in FIQ reductions in relation to duration of the condition (mean reduction of 16.72, 16.00 and 16.12 for < 9 years, and 19.38, 12.09 and 14.50 for ≥ 9 years at eight weeks and four and six months, *P *= 0.52, 0.38 and 0.73, respectively).

### Clinically meaningful outcomes

Minimal clinical differences for pain (30% reduction from baseline) [21] and FIQ (14% reduction from baseline) [25] have been established. The number of individuals with a ≥ 2 point reduction in NRS-PI ranged from 38 to 51% in the combined CFQ group, and this was significantly higher than in the usual care group (9 to 18%) at all times (Table 3A). Those with a ≥ 8.8 unit reduction in FIQ scores ranged from 56 to 71% in the combined CFQ group, and this was also significantly higher than in the usual care group (20 to 34%) at all times (Table 3B). A reduction in PSQI of ≥ 3 is considered a treatment effect [26]. In the combined CFQ group, 48 to 51% attained this, compared to 22 to 25% in the control group (Table 3C). In the CFQ group, 33 to 36% attained an increase of ≥ 6.5 points in SF-physical scores compared to 9 to 14% in the control group; for SF-mental scores, 30 to 38% in the CFQ group attained an increase of ≥ 7.9 points compared to 11 to 27% in the control group (Table 3D, E). These latter differences are considered clinically meaningful in fibromyalgia [12].

**Table 3:** Individuals with clinically meaningful improvements

Variable	Usual care	Combined qigong	*P*-value*
**A. NRS-PI^1^**			
**2 months**	4/45 (8.9%)	36/76 (47.4%)	*P *< 0.0001
**4 months**	7/44 (15.9%)	37/73 (50.7%)	*P *= 0.0002
**6 months**	8/44 (18.2%)	28/73 (38.4%)	*P *= 0.02
**B. FIQ^2^**			
**2 months**	9/45 (20.0%)	53/75 (70.7%)	*P *< 0.0001
**4 months**	13/44 (29.5%)	41/73 (56.2%)	*P *= 0.005
**6 months**	15/44 (34.1%)	41/73 (56.2%)	*P *= 0.02
**C. PSQI^3^**			
**2 months**	11/45 (24.4%)	39/76 (51.3%)	*P *= 0.004
**4 months**	10/44 (22.7%)	35/73 (47.9%)	*P *= 0.007
**6 months**	11/44 (25.0%)	36/73 (49.3%)	*P *= 0.01
**D. SF-36 physical^4^**			
**2 months**	4/45 (8.9%)	27/76 (35.5%)	*P *= 0.001
**4 months**	5/44 (11.4%)	24/73 (32.9%)	*P *= 0.009
**6 months**	6/44 (13.6%)	24/73 (32.9%)	*P *= 0.02
**E. SF-36 mental^4^**			
**2 months**	5/45 (11.1%)	29/76 (38.2%)	*P *= 0.001
**4 months**	6/44 (13.6%)	22/73 (30.1%)	*P *= 0.05
**6 months**	12/44 (27.3%)	26/73 (35.6%)	*P *= 0.35

* *P*-values calculated by using the chi-square test

^1 ^Reduction of ≥ 2 points (30%) from baseline [21]

^2 ^Reduction of ≥ 8.8 points or 14% from baseline [25]

^3 ^Reduction of ≥ 3 PSQI points considered a treatment response [26]

^4 ^Increase of 6.5 points (SF-physical) and 7.9 points (SF-mental) [12]

### Relationship of outcomes to extent of practice

**Figure 5:**
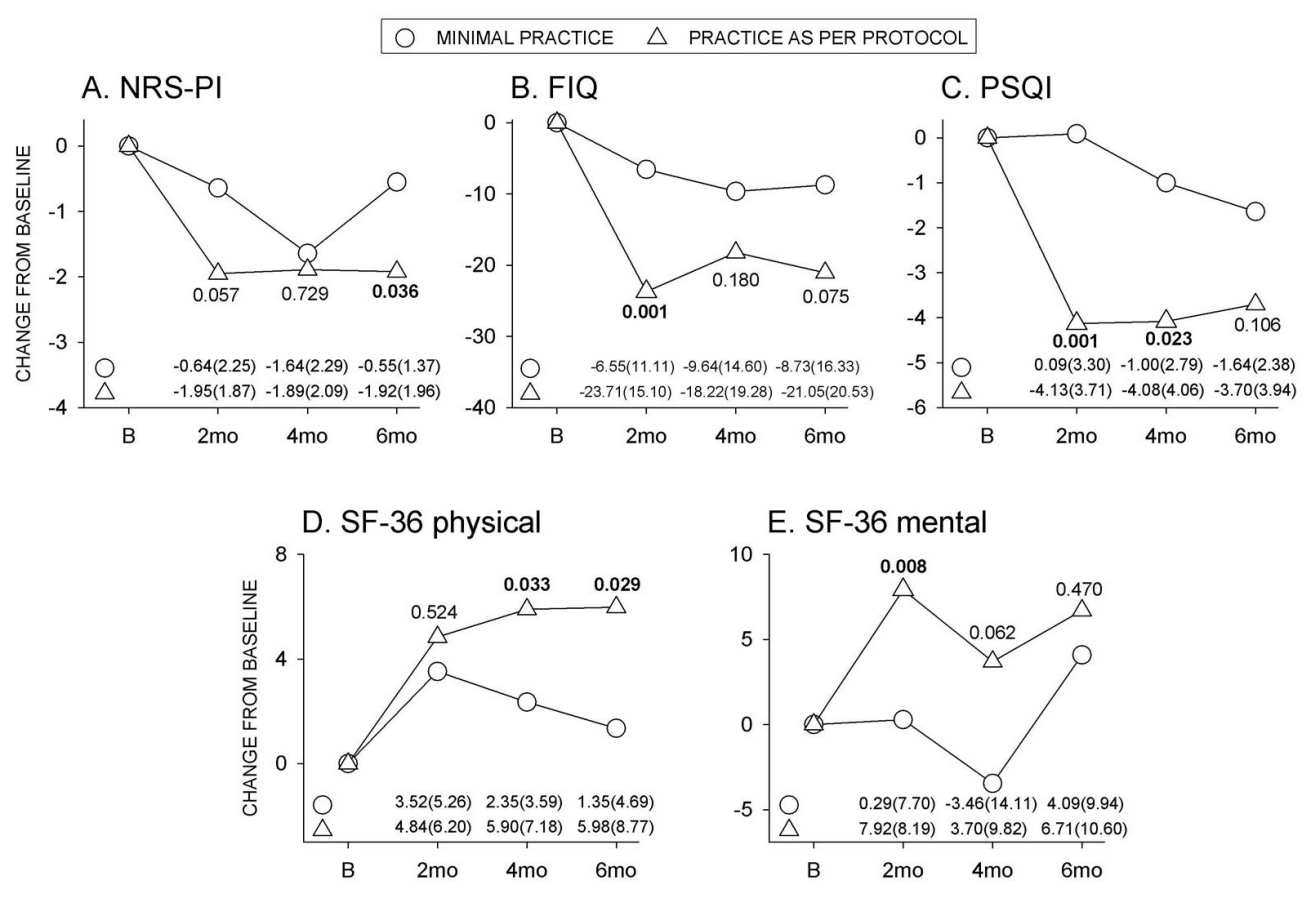
**Comparison of outcomes in the per protocol practice group and those who practiced minimally**. Values are means for those who practiced minimally (≤ 3 h/week, *N *= 11), and those who practiced as per protocol (≥ 5 h/week, *N *= 38). Numbers at the bottom of the figure indicate mean (SD) values. Between-group *P*-values indicated within figure. B, baseline; mo, month.

The design of this trial included CFQ instruction and a commitment to a minimum of 45 minutes daily practice for eight weeks. We recognize this was a significant commitment and that not all would be able to meet this goal, despite best intentions. Self-reports of weekly practice times indicated 38/73 (52%) practiced for ≥ 5 h per week for this interval, which represents per protocol practice. There was also a cohort who completed training but then practiced minimally (≤ 3 h/week) (*N *= 11/73; 15%). Figure 5 presents a comparison of outcomes for those who adhered to the protocol and those who practiced minimally. There were significantly greater improvements in pain (six months, Figure 5A), FIQ (two months, Figure 5B), PSQI (two and four months, Figure 5C), physical function (four and six months, Figure 5D) and mental function (two months, Figure 5E) in the per protocol group. Within the per protocol group, 53 to 57% had a ≥ 2 point reduction in NRS-PI, 60 to 82% had a reduction of ≥ 8.8 units in FIQ, and 55 to 65% had a drop of ≥ 3 units in PSQI scores; furthermore, 37 to 43% had increased SF-physical scores of ≥ 6.5 and 32 to 42% had increased SF-mental scores of ≥ 7.9 over the six-month duration of the study.

### Adverse events

Two participants experienced adverse events judged to be possibly related to the CFQ practice. One patient experienced an increase in right-sided shoulder pain, and another participant experienced an episode of plantar fasciitis. In both cases, patients were able to continue with the study and the pain settled over time.

## Discussion

The current randomized controlled trial demonstrates that CFQ, a particular form of qigong, is potentially a useful self-care practice for individuals with fibromyalgia. Improvements in pain, FIQ, sleep and physical and mental function were observed using well-validated measures, and improvements observed at the end of the training and committed-practice interval were maintained in the longer term at four months and six months. The design of this trial included two different training cohorts separated by an interval of six months; outcomes in both groups were similar over time and this indicates the essential reproducibility of findings in two different groups with fibromyalgia.

Results in this trial, where improvements in fibromyalgia symptoms persist in the longer term for four to six months, are generally consistent with recent controlled trials of qigong [11] and tai chi [12], both of which are regarded as "meditative movement" [13]. Each of these studies attended to the need to engage in regular practice following training, and report more robust improvements than earlier trials where practice was limited [14-16]. With these practices, the issue of how much practice is required to see benefit (threshold effect) and to sustain benefit (produce long-term changes) requires attention. In the present trial, significant and sustained benefits were observed in the combined group even though only 38/73 (52%) of those who completed the study self-reported practice as per protocol for the initial eight weeks. A separate analysis of those who actually completed per protocol practice (≥ 5 h/week) indicates a significantly greater improvement in pain, FIQ, PSQI and physical and mental function at certain time intervals compared to those who engaged in minimal practice. As with other forms of self-practice, understanding the factors that determine motivation for engaging in, and continuing with, self-practice will be important for elaborating the potential for these techniques to be of benefit for those with fibromyalgia.

There is a significant and emerging body of evidence for health benefits of qigong and tai chi in a variety of conditions [27]. These practices are complex, multi-component interventions with inherent research challenges [28,29]. Both practices encompass two broad domains, movement and mind-body approaches, and these domains potentially can be considered separately. There is an extensive literature on exercise in fibromyalgia, and much of this (75% of studies) emphasizes aerobic exercise and combined exercise, strength and flexibility activity [30]. Meta-analyses and systematic reviews indicate that exercise can provide benefit in fibromyalgia, although there are differences in conclusions relating to intensity of practice needed to observe the benefit [6,31,32]. It is important to recognize that the movement involved in CFQ, the form of qigong evaluated in the present study, is loose and gentle and does not involve aerobic elements, and may be fundamentally different from aerobic exercise. There is also a developing literature on mind-body practices, such as mindfulness-based stress reduction (MBSR) and meditation for fibromyalgia. Preliminary studies reported some benefit in fibromyalgia using these approaches [33-35], but a recent more extensive controlled trial reported limited benefit with MBSR [36]. Comparative trials, in which cohorts of patients undertake qigong versus exercise, or qigong versus mind-body modalities, will be required in order to ascertain whether qigong produces benefits that differ from these other approaches.

### Strengths and limitations of study

There are several notable features to this study, including the recruitment of adequate numbers, the inclusion of two cohorts (immediate, delayed training groups) which allows for assessment of reproducibility between groups, the use of standardized measures to determine outcomes, high follow-up rates for those who complete training, long-term outcomes to six months, and attention to adherence to protocol with *post-hoc *analysis in relation to the amount of practice. In addition, there is also a consideration of those who attain outcomes that are considered to represent clinically meaningful changes. There are also limitations. (1) The study was not blinded. With a complex intervention involving movement and mind-body elements, it is not possible to blind trials. Comparisons with other groups in which specific elements are engaged are possible, and this may be the most pragmatic approach for elaborating the therapeutic potential of qigong for fibromyalgia. (2) The control group was a wait-list group that underwent usual care. As such, they did not receive attention or engage in movement as did the intervention group, and these elements may contribute to outcomes. Clinical practice includes many non-specific elements which can contribute to the overall efficacy of a treatment. With a complex health condition, where symptoms persist despite previous treatment regimes, the most relevant consideration may be the number of patients who attain clinically meaningful reductions in symptoms. Within this study, significant numbers of those who completed CFQ training and practice attained benchmarks both when compared to the wait-list group, and when considered in absolute terms for the per protocol group. (3) This study evaluated efficacy of qigong in those who completed the study. The main study question was the efficacy of CFQ for fibromyalgia over six months when practiced diligently for eight weeks. A total of 81% of both groups who undertook training completed the six-month study, and this is a respectable rate of completion given practice requirements. The *post-hoc *analysis in relation to the extent of practice indicates less benefit was derived with minimal practice, so if one includes those who did not complete the practice in the analysis, a reduced effect would be observed. It is not possible to evaluate the effect of a technique if it is not implemented, and other forms of analysis will not address the essential study question. (4) This study examined one particular form of qigong and the generalizability of results is not known. CFQ was elaborated and introduced into North America in the 1990s [19]. There are many forms of qigong, with references to hundreds of different forms, as well as considerations of common elements [13,27,37]. A study conducted in Sweden using a different form of qigong has reported sustained beneficial effects in fibromyalgia [11], and there are case reports from the United States of benefits in fibromyalgia with external qigong [38]; these observations support the involvement of common elements. Given the multiplicity of forms and their geographic representations, research into the medical potential of qigong will need to attend both to common elements, as well as to exploring whether particular forms of qigong are more effective than others.

## Conclusion

Findings reported in this study indicate that self-practice of CFQ, a particular form of qigong, leads to long-term beneficial effects in fibromyalgia in several domains and may be a useful adjunct in the management of fibromyalgia. Further study of the potential health benefits of this modality is warranted.

## Abbreviations

B: baseline; BMI: Body Mass Index; CFQ: Chaoyi Fanhuan Qigong; FIQ: fibromyalgia impact questionnaire; IMMPACT: Initiative on Methods, Measurement, and Pain Assessment in Clinical Trials; IQR: interquartile range; MANOVA: multivariate analysis of variance; MBSR: mindfulness-based stress reduction; mo: month; NRS-PI: numerical rating scale pain intensity; PSQI: Pittsburgh Sleep Quality Index; SD: standard deviation.

## Competing interests

ML and JS have no competing interests relevant to this study. CH and DM are community-based CFQ instructors. CH is coauthor of several books on CFQ.

## Authors' contributions

ML and JS conceived and designed the trial, analyzed and interpreted data, and drafted the manuscript. CH conducted the initial CFQ training sessions. DM conducted the weekly follow-up sessions for eight weeks. All authors have read and approved the manuscript for publication.
